# Testicular cancer in Latvia: incidence trends and prognostic outcomes across a 23-year study

**DOI:** 10.1186/s12885-025-14792-8

**Published:** 2025-10-01

**Authors:** Klims Leonenko, Eva Petrosina, Maksism Zolovs, Janis Auzins, Igors Andzans, Vilnis Lietuvietis, Ilze Konrade

**Affiliations:** 1 Riga Stradiņš University, Riga, Latvia; 2 Daugavpils University, Daugavpils, Latvia; 3 Riga East University Hospital, Riga, Latvia

## Abstract

**Background:**

The aim of this study was to analyze trends in testicular cancer incidence, mortality, and survival in Latvia from 1994 to 2017.

**Materials and methods:**

This retrospective cohort study analyzed testicular cancer patients diagnosed in Latvia between 1994 and 2017. The population-based cancer registry recorded nearly all cases. Patients were categorized by age, tumor histology (seminoma, non-seminoma, unspecified), and disease stage according to the TNM classification system.

The Joinpoint regression model was used to analyze trends in incidence, mortality. Kaplan-Meier analysis was performed to calculate 5- and 10-year cancer-specific and overall survival probabilities.

**Results:**

During the study period, the age-standardized incidence rate of testicular cancer increased from 2.25 to 3.57 per 100,000, with an annual percentage change (APC) of 2.37% (95% CI: 0.78, 3.9).

The incidence of both seminoma and non-seminoma increased significantly. The APC for seminoma was 3.186% (95% CI: 0.429, 5.991), while for non-seminoma, it was 4.104% (95% CI: 0.486, 52.597).

The overall and cancer-specific 5-year survival probabilities in Latvia were 70.6% (95% CI: 67, 74) and 76.4% (95% CI: 73.4, 79.6), respectively. The overall and cancer-specific 10-year survival probabilities were 66% (95% CI: 62, 69) and 74.0% (95% CI: 70.8, 77.4), respectively.

The 5-year overall survival rate increased by 19.8 percentage points, from 59.1% in 1994-1999 to 80.9% in 2010-2017. The observed 5-year cancer-specific survival increased by 27.4 percentage points over the same period.

The mortality rate did not change significantly, with an APC of 1.32% (95% CI: -1.52 to 4.2) during this period.

**Conclusions:**

A moderate increase in testicular cancer incidence was observed in Latvia over 23 years, while the mortality rate remained stable. The 5-year relative survival improved over different time periods; yet outcomes could be further enhanced if a multidisciplinary approach to diagnostics and management had been implemented in Latvia, as in other countries.

## Introduction

Testicular cancer accounts for approximately 5% of all urological malignancies. However, it is the most common malignant tumour in young men aged 15–45 years, who represent a key socioeconomically active demographic [1, 2].

Over recent decades, the incidence of testicular cancer has been steadily increasing worldwide, with significant variation across countries [3, 4]. The highest incidence rates are reported in highly industrialized nations [3–7], with Norway showing the highest recorded rate at 11.5 cases per 100,000 men. In comparison, Latvia has a relatively lower incidence rate of 3.5 cases per 100,000 men [3, 8]. Nonetheless, this is the highest rate among the Baltic states.

The exact aetiology of testicular cancer remains unclear. However, several established risk factors have been identified, including components of testicular dysgenesis syndrome [9–11]. Additionally, recent genetic studies have contributed to a deeper understanding of disease susceptibility, for example, Pluta et al. identified 22 genomic loci associated with an increased risk of testicular cancer [12].

Despite the rising incidence, testicular cancer is highly curable. Advances in chemotherapy—particularly the introduction of platinum-based regimens—have led to overall survival rates exceeding 90% [13].

The aim of this study was to analyse trends in incidence, mortality, and survival of testicular cancer in Latvia, and to place these findings in the context of regional and global patterns.

## Materials and methods

This retrospective cohort study included patients who were diagnosed with testicular cancer based on the International Classification of Diseases (ICD-10, C62) between January 1, 1994, and December 31, 2017. Since 1963, Latvia has had population-based cancer registry that covers the entire country (territory: 64 589 km^2^; population: 1.94 million as of January 1, 2017) [14]. The registry records approximately 100% of all cancer cases diagnosed [15].

This study specifically focused on germ cell tumors (GCTs) of the testis. In accordance with our predefined inclusion criteria, we systematically excluded cases with non-germ cell testicular tumors (including but not limited to Leydig cell tumors, Sertoli cell tumors, and other sex cord-stromal tumors) to maintain diagnostic homogeneity within our study cohort.

For the analysis, patients were divided into subgroups based on their age at diagnosis (< 25, 25–34; 35–44; 45–64; >65), tumour histology, and stage of the disease. The histology of the tumours was classified using the International Classification of Diseases for Oncology, 3rd edition (ICDO-3) codes. The histology codes were grouped into free categories: seminoma (ICDO-3 codes: 9061–9064), non-seminoma (ICDO-3 codes: 9065, 9070–9072, 9080–9085, 9100–9102), and unspecified. The stage of the disease was determined based on the TNM classification system.

All analyses were performed using RStudio, R Statistical Software (V4.2.3; R Core Team 2023. Descriptive analysis was performed. Categorical variables were expressed as counts and relative counts. Continuous variables were expressed as means and standard deviations.

The incidence and mortality rates of testicular cancer in the Latvian population were calculated by determining the number of cases per 100,000 men based on data obtained from the Central Statistical Bureau of Latvia. Then, age-standardized (AS) incidence and mortality rates were calculated from age-specific rates by weighting directly to the World Population Standard and the World Health Organization Standard Population in 2000. Age-standardized rates were calculated for all ages combined, histology categories, and age groups.

The rates of incidence and mortality were modelled using Joinpoint regression analysis, which enabled the calculation of the annual percentage change (APC) with 95% confidence interval (CI) to determine the trends in incidence and mortality over time. The APC was significantly different from zero (*p* < 0.05).

The Joinpoint Regression Analysis programme (version 4.6.0.0, April 16, 2018, USA) was used.

The patients were divided into three distinct time periods: 1994–1999, 2000–2009 and 2010–2017.

Patients were followed up from the date of diagnosis until death, censoring, or December 31, 2017.

We utilized Kaplan‒Meier analysis to calculate the 5-year, 10-year, and overall cancer-specific survival probabilities.

All *p* values less than 0.05 were considered to indicate statistical significance.

## Results

**Fig. 1:**
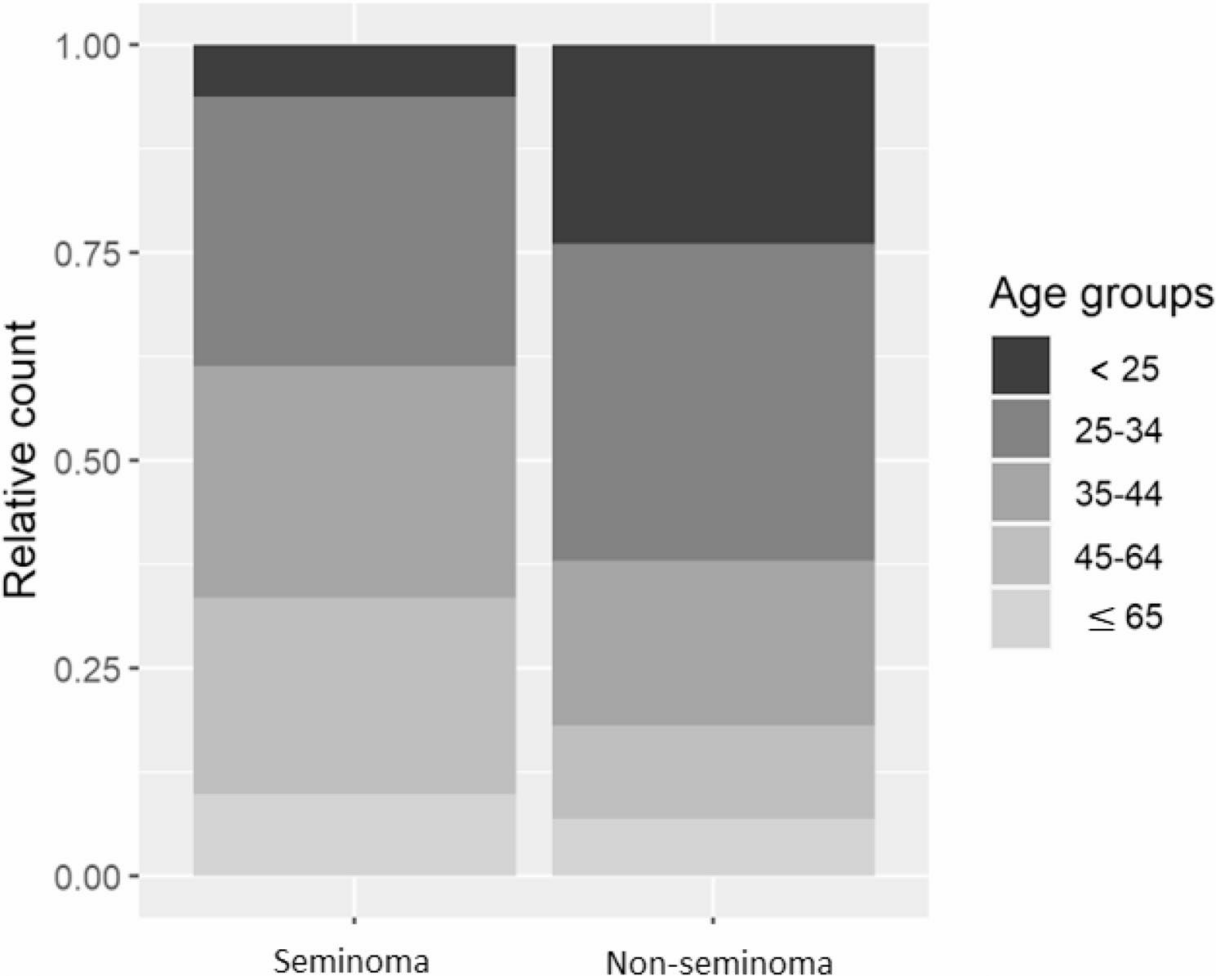
Seminoma and non-seminoma distribution by age groups

Overall, 786 new testicular cancer cases were diagnosed between 1994 and 2017 across all age groups. On average, 31 cases were diagnosed each year during this 23-year period. Most of the patients were 25–34 years of age (266) at diagnosis, followed by 35–44 years of age (186) and 45–65 years of age (144). Only 15 patients were younger than 18 years, from 16 to 18 years old, and 83 patients were older than 65 years (Table 1). Compared with seminomas, non-seminomas were more common in younger patients and less common in older patients (Fig. 1). Seminoma was diagnosed in 406 patients, non-seminoma was diagnosed in 309 patients, and unspecified cancers were diagnosed in 71 patients. The majority of testicular cancer cases were diagnosed in patients residing outside the capital city of Riga, especially patients who resided in small towns and rural areas (637), followed by large towns (117) and the capital city (32).

**Table 1:** Main characteristics of testicular cancer patient cohort by time period in Latvia 1994–2017

Characteristic	Overall, *N* = 786^1^	1994–1999, *N* = 167^1^	2000–2009, *N* = 340^1^	2010–2017, *N* = 279^1^
Morphology				
Seminoma	406 (56.8%)	84 (56.0%)	177 (56.9%)	145 (57.1%)
Non-seminoma	309 (43.2%)	66 (44.0%)	134 (43.1%)	109 (42.9%)
Unknown	71	17	29	25
Clinical Stage				
Stage I	331 (47.6%)	64 (45.4%)	136 (44.6%)	131 (52.4%)
Stage II	168 (24.1%)	20 (14.2%)	83 (27.2%)	65 (26.0%)
Stage III	197 (28.3%)	57 (40.4%)	86 (28.2%)	54 (21.6%)
Unknown	90	26	35	29
Age group, years				
< 25	107 (13.6%)	21 (12.6%)	47 (13.8%)	39 (14.0%)
25–34	266 (33.8%)	63 (37.7%)	112 (32.9%)	91 (32.6%)
35–44	186 (23.7%)	34 (20.4%)	73 (21.5%)	79 (28.3%)
45–64	144 (18.3%)	23 (13.8%)	68 (20.0%)	53 (19.0%)
≥ 65	83 (10.6%)	26 (15.6%)	40 (11.8%)	17 (6.1%)
Place of residence				
Capital	32 (4.1%)	19 (11.4%)	8 (2.4%)	5 (1.8%)
Big towns	117 (14.9%)	22 (13.2%)	55 (16.2%)	40 (14.3%)
Region	637 (81.0%)	126 (75.4%)	277 (81.5%)	234 (83.9%)

^1^n (%)

**Fig. 2:**
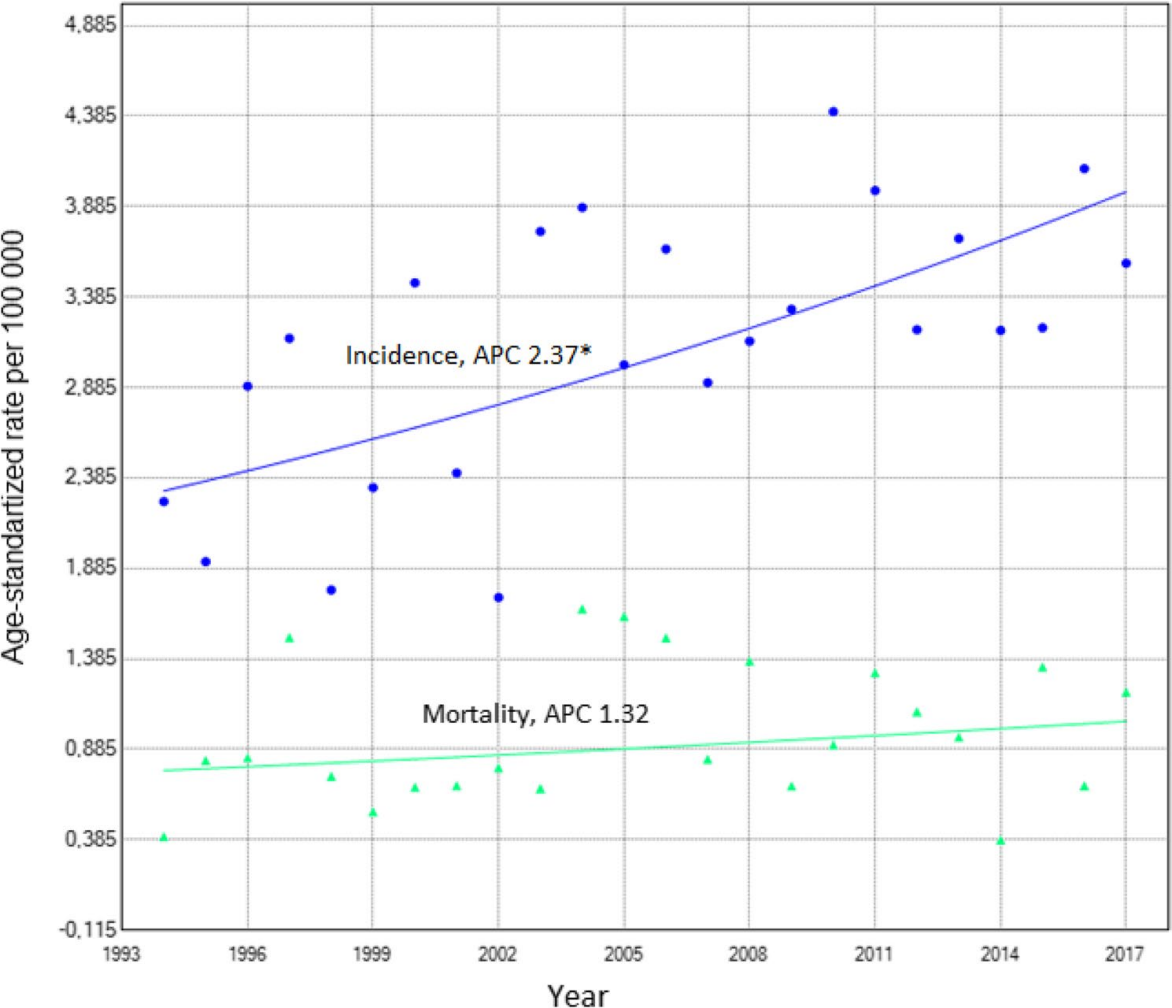
Testicular cancer incidence and mortality trends using Joinpoint analysis. APC – annual percentage of change; * represents statistically significant change

The overall incidence of testicular cancer increased from 1994 to 2017 (APC = 2.373% (95%CI −0.78, 3.94)), but mortality rate did not change significantly (APC 1.32% (95% CI −1.52 to 4.2)) (Fig. 2). In the 35–44 years age group, the incidence annual percent change (APC) significantly increased until 2015, with an APC of 5.893 (95% CI-3,645, 11,230). However, after 2015, the APC decreased significantly to −44.507 (95% CI −62.429, −1.472). The APCs of the incidence of both seminoma and non-seminoma exhibited statistically significant increases. The APC of the incidence of seminoma was 3.186 (95% CI: 0.429, 5.991), while the APC of the incidence of non-seminoma was 4.104 (95% CI: 0.486, 52.597) until 2011.

The incidence of stage I disease significantly increased, with an APC of 3.556 (95% CI 2.083, 5.051). Regarding Stage II, there was one change point in 2003. During the period from 1994 to 2003, the APC was 18.974 (95% CI 6.764, 135.191), but during the period from 2003 to 2017, there were no statistically significant changes.

The total overall and cancer-specific 5-year survival rates for patients with testicular cancer in Latvia were 70.6% (95% CI: 67, 74) and 76.4% (95% CI: 73.4, 79.6), respectively, between 1997 and 2017. Moreover, the overall and cancer-specific 10-year survival rates were 66% (95% CI: 62, 69) and 74.0% (95% CI: 70.8, 77.4), respectively.

Analyzing the 5-year overall survival trends by age groups did not reveal any significant variations. For patients younger than 25 years, the survival rate was 74.1% (95% CI: 65.9, 83.3); aged 25–34 years, it was 79.3% (95% CI: 74.5, 84.4); aged 35–44 years, it was 82.1% (95% CI: 76.7, 88.0); aged 45–64 years, it was 64.1% (95% CI: 56.4, 72.9); and over 65 years, it was 24.5% (95% CI: 16.7, 35.8).

During the study period, data shows a positive trend in overall survival probabilities over the decades. In 1994–1999, 5-year survival was 59.1% (95 CI: 52.1, 67.1); in 2000–2009 it was.

**Fig. 3:**
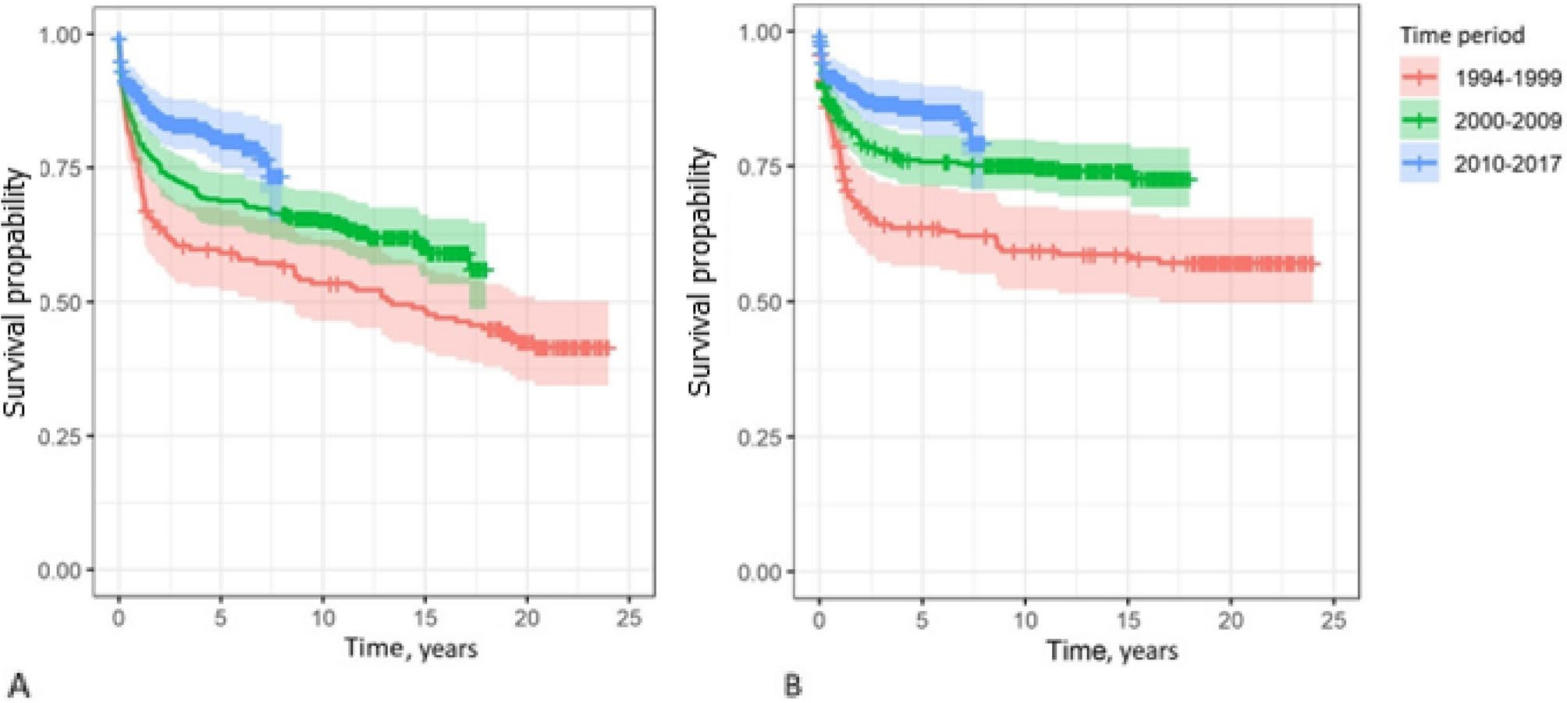
Overall (**A**) and cancer specific (**B**) testicular cancer survival by three time periods

68.8% (95 CI: 64.1, 73.9) and in 2010–2017 it was 80.9% (95 CI: 76.0, 86.1) (Fig. 3A; Table 2). The 5-year overall survival rate increased by 19.8% points from 59.1% in years 1994–1999 to 80.9% in years 2010–2017. Observed 5-year cancer specific survival increased by 27.4% points from 63.5% in years 1994–1999 to 80.9% in years 2010–2017 (Fig. 3B; Table 2).

**Table 2:** 5-year and 10-year survival rate (%) in the three time periods

Survival rate		1994–1999	2000–2009	2010–2017
5-year (95% CI)	OS CSS	59.1 (52.1, 67.1) 63.5 (56.5, 71.4)	68.8 (64.1, 73.9) 75.8 (71.3, 80.6)	80.9 (76.0, 86.1) 86.0 (81.7, 90.5)
10-year (95% CI)	OS CSS	53.4 (46.3, 61.6) 65.2 (60.3, 70.5)	65.2 (60.3, 70.5) 75.2 (70.6, 80.0)	

*CI *Confidence interval, *CSS *Cancer specific survival, *OS *Overall survival

The five-year specific survival rates were 91.7% for localized stage I tumours, 89.2% for stage II tumours, and 41.9% for stage III tumours. We did not observe a statistically significant difference in cancer morphology, as the survival rate for seminoma was 81.3% (95% CI: 77.4–85.3), which was comparable to that for non-seminoma patients (75.8% 95% CI: 70.9–81%; *p* > 0.05). There was a significant difference in survival rates between age groups (*p* < 0.001), with group aged 64 and older having a shorter survival time than the other age groups.

We analyzed overall survival by residential area and found no statistically significant differences between urban (64%, 95% CI: 0.56–0.72) and rural areas (72%, 95% CI: 0.69–0.76) for 5-year survival, and 62% (95% CI: 0.62–0.70) vs. 66% (95% CI: 0.66–0.71) for 10-year survival, respectively (*p* = 0.156).

However, survival probabilities appeared lower in urban districts. The 5-year survival probability in urban districts was 69% (95% CI: 0.61–0.77), compared to 78% (95% CI: 0.75–0.82) in rural districts. Similarly, the 10-year survival probability was 68% (95% CI: 0.60–0.76) in urban areas versus 76% (95% CI: 0.72–0.79) in rural areas.

## Discussion

In our study, we analysed trends in the incidence of seminoma and non-seminoma testicular cancer in Latvia from 1994 to 2017. We observed similar incidence and mortality trends as those reported in Scandinavian, Western European and other Baltic countries. Furthermore, increasing survival rates were observed.

Testicular cancer incidence remains highest in Northern Europe, with age-standardized rates of 11.7 in Norway and 11.2 per 100,000 individuals in Denmark [3, 16]. However, trends show only modest increases, with an APC of 2.4% (95% CI: 2.0–2.8) in Norway and 0.8% (95% CI: 0.4–1.3) in Denmark, indicating relatively stable patterns [3].

In contrast, countries in Central and Eastern Europe, where incidence rates are lower, have shown more pronounced increases, with APCs ranging from 3 to 5%. Our analysis revealed a similar trend in Latvia, with an incidence of 3.57 per 100,000 and an APC of 2.37% (95% CI: 0.78–3.94). Comparable results were observed in Lithuania (incidence 3.45 per 100,000; APC 2.97%, 95% CI: 0.9–5.1) [17–20].

This study demonstrates a five-year survival rate of 76% for Latvia, which is comparable to neighbouring countries—67% in Lithuania and 74.5% in Estonia [5, 11]. Our analysis also revealed a positive trend over time, with five-year survival increasing from 68.8% in 2000–2009 to 80.9% in 2010–2017, what reflects advancements in both treatment and diagnostic strategies. In a study conducted by Trama et al. based on EUROCARE-5 data, it was found that the five-year relative survival rate for patients with testicular cancer was 89% [19]. However, when considering age-standardized five-year survival rates across different parts of Europe (Northern, Central, Southern, and Eastern), the rate ranged from 80 to 92%.

Our findings indicate that survival curves for testicular cancer patients stabilized approximately 2–3 years post-diagnosis, suggesting durable long-term survival. Notably, five- and ten-year survival rates were nearly identical, differing by only around 5%.

This phenomenon suggested that once patients surpassed the initial critical period following diagnosis and treatment, the long-term survival outlook remained relatively consistent between the five-year and ten-year follow-ups. This finding underscores the importance of early detection and effective treatment for improving the overall prognosis of individuals diagnosed with testicular cancer.

Gurney et al. reported that in the majority of countries with available histological data, there was a higher incidence of seminoma than non-seminoma. Furthermore, both types of testicular cancer showed an increasing trend over time. The proportion of seminomas in Latvia is 56%, which is consistent with trends observed in Central European countries and slightly higher than in neighboring countries—46% in Lithuania and 37% in Estonia, respectively [17, 19–23]. Although both types have demonstrated upward trends in several high-incidence countries, seminomas have sometimes increased at a faster pace [3].

Regarding temporal patterns, seminoma and non-seminoma incidence rates have generally followed similar trends. However, some high-incidence countries show slight divergence in recent years [24]. In our analysis, both subtypes increased, with APCs of 4.1% (95% CI: 0.48–5.2) for seminomas and 3.18% (95% CI: 0.42–5.9) for non-seminomas. These trends are consistent with patterns observed in neighboring and other European countries [17, 19, 21] and confirm European data that seminomas are typically diagnosed around 10 years later (ages 35–40) than non-seminomas (around age 25) [3].

The observed age differences between seminomas and non-seminomas may be explained by distinct tumour biology. Non-seminomas typically demonstrate more aggressive behaviour, rapid progression, and a higher likelihood of metastatic presentation at diagnosis, contributing to their earlier age distribution [25].

Trama et al. reported five-year relative survival rates in Europe of 93.9% for seminoma and 88.3% for non-seminoma, underscoring the prognostic impact of histology [19]. The highest survival rates were observed in Northern Europe, reaching 97.7% for seminoma and 90.2% for non-seminoma.

In our study, we observed that the five-year cancer-specific survival rates were lower than reported in other countries and regions, 81% for seminomas and 76% for non-seminomas. These outcomes are also less favourable compared to Lithuania, where the five-year survival rates were 93.98% and 87.94%, respectively [21].

In our study, the majority of testicular cancer cases were observed among individuals from rural areas, aligning with findings by Sonneveld et al. [26]. While various environmental and hormonal factors have been proposed, only a few have shown consistent associations with increased testicular cancer risk [27]. This location pattern may be explained by a relatively high frequency of shared genetic traits among individuals with common ancestry in these regions, including genes potentially associated with testicular cancer susceptibility. While definitive causal relationships for many environmental exposures remain unconfirmed, prenatal exposure to endocrine-disrupting chemicals—such as pesticides (e.g., DDT), phthalates, polychlorinated biphenyls (PCBs), and bisphenol A (BPA)—is currently not considered a major concern in Latvia, given the country’s predominantly agrarian structure and limited industrial activity involving these substances [28, 29].

In our study, five-year cancer-specific survival was higher in rural areas compared to urban areas, at 78% and 69% respectively. This contrasts with findings from neighbouring countries such as Belarus, where survival rates were reported at 79% in urban areas and only 59% in rural areas [30]. Previous research on genitourinary tract cancers has suggested that better access to medical services in urban settings typically correlates with improved survival outcomes [31–34].

One possible explanation for the observed trend in Latvia may be the effectiveness of national healthcare programs that ensure timely access to diagnostics and treatment for cancer patients, even in rural areas. Additionally, rural environments may offer cleaner ecological conditions with lower exposure to environmental risk factors, which could positively influence cancer progression and survival.

Identifying early trends in rural versus urban cancer outcomes is important for designing targeted healthcare interventions. This is particularly relevant for underserved or vulnerable populations, where access to timely care may be inconsistent. Focused public health strategies can help reduce disparities and improve cancer survival across different geographic regions.

As demonstrated in our study, better survival rates were observed in younger age groups, particularly those under 45 years, with a five-year survival rate of 82.1%, consistent with findings by Møller et a l [25]. This aligns with the EUROCARE-5 data, which reported a five-year relative survival rate of 96.5% for individuals aged 15–39 years and impressive 90% for individuals older than 40 years [19].

The analysis by Fossa et al. presented intriguing findings, revealing an adverse impact of increasing age on testicular cancer-specific mortality [35]. The researchers proposed a hypothesis suggesting that the combination of reduced treatment intensity and heightened therapy-related toxicity might be a plausible explanation for the increased testicular carcinoma-specific mortality in patients older than 40 years. This study sheds light on the complex interplay of various factors that can influence the prognosis of older individuals with testicular cancer.

Physiological decreases in bone marrow capacity and kidney function [36], as well as a greater incidence of life-threatening conditions, such as second malignancy and cardiovascular disease, are observed among individuals who have survived testicular cancer than in the general population [37, 38].

The variation in five-year relative survival rates between our study and other Nordic and Baltic countries indicates that a meticulous analysis of the situation should be conducted. Several potential factors may explain these differences. One significant aspect is the presence of diagnostic uncertainty, which may result from limited access to advanced diagnostic tools. Additionally, the accessibility of urologists, particularly outside major cities, could contribute to delayed medical consultations and diagnoses. A concerning finding from our study is that nearly half of the patients presented with metastatic disease in clinical stage II and III, shedding light on the importance of early detection and prompt medical intervention.

Testicular cancer, a rare and malignant neoplasm, accounts for only 5% of all urologic diseases, with approximately 30 cases reported annually in Latvia. These patients are dispersed across various hospitals, leading to potential variations in treatment approaches and sometimes inadequate time intervals between diagnosis and treatment, especially for those with metastatic disease. Numerous studies emphasize the importance of centralizing the treatment of rare pathologies in a single clinical centre, where a comprehensive and multidisciplinary approach can be employed. Such an approach ensures that patients receive standardized and optimal care, enhancing their chances of better outcomes and improved quality of life. The need for a more unified and coordinated approach for managing testicular cancer patients is paramount, and it underscores the significance of centralizing care to achieve the best possible results [39–41].

Since the introduction of cisplatin-based medicine for the treatment of testicular cancer, this once formidable disease has transformed into one of the most curable types of cancer. In developed countries, remarkable progress has been made in improving surveillance, leading to impressive five-year survival probabilities of up to 95% and a significant reduction in cancer-specific mortality. However, our study, which focused on Latvia, reveals a contrasting observation. Despite advancements in surveillance and treatment, the mortality rate in Latvia has not significantly changed. The average annual percentage change in the APC was 1.32 (95% CI −1.525 to 4.2) for cancer-specific mortality in Latvia.

Therefore, it is crucial to conduct long-term monitoring and follow-up of testicular cancer survivors. Population-based cancer registries can serve as valuable resources for conducting survivorship studies to better understand the health outcomes and long-term effects of testicular cancer and its treatments [42].

The imperative to enhance information, popularize the topic on social media, and improve education on cancer risk groups and self-examinations is of utmost importance—particularly for young boys and their parents—as it may contribute to earlier tumor detection. As stated in the insightful study McGuinness et al. [43], increasing awareness about cancer risk factors and empowering individuals with knowledge about self-examinations can be instrumental in early detection and prevention efforts. This concerted effort to improve awareness and education can pave the way for a healthier and more informed generation. Additionally, the education of general practitioners and the increased availability of testicular ultrasound in smaller hospitals and outpatient clinics—particularly in regions outside the capital—can ultimately contribute to earlier detection, improved cancer outcomes, and enhanced overall well-being.

## Conclusion

Testicular cancer primarily affects young men, and although significant improvements have been in other Northern European and Baltic countries, there is still a need to enhance men’s access to urologists, regardless of their location. Given that testicular cancer primarily affects young men, it is crucial to prioritize initiatives that provide modern, comprehensive, and multidimensional approaches to diagnosis, treatment, and long-term follow-up. Ensuring optimal care not only improves survival but also preserves quality of life, which is especially vital for this patient group.

## Data Availability

All data generated or analysed during this study are included in this published article.The datasets used and/or analyzed during the current study can be made available by the corresponding author on reasonable request.
